# Primer Based Approach for PCR Amplification of High GC Content Gene: *Mycobacterium* Gene as a Model

**DOI:** 10.1155/2014/937308

**Published:** 2014-03-24

**Authors:** Arbind Kumar, Jagdeep Kaur

**Affiliations:** Department of Biotechnology, Panjab University, Sector 14, Chandigarh 160014, India

## Abstract

The genome of *Mycobacterium* is rich in GC content and poses problem in amplification of some genes, especially those rich in the GC content in terminal regions, by standard/routine PCR procedures. Attempts have been made to amplify three GC rich genes of *Mycobacterium* sp. (*Rv0519c* and *Rv0774c* from *M. tuberculosis* and *ML0314c* from *M. leprae*). Out of these three genes, *Rv0774c* gene was amplified with normal primers under standard PCR conditions, while no amplification was observed in case of *Rv0519c* and *ML0314c* genes. In the present investigation a modified primer based approach was successfully used for amplification of GC rich sequence of *Rv0519c* through codon optimization without changing the native amino acid sequence. The strategy was successfully confirmed by redesigning the standard primers with similar modifications followed by amplification of *ML0314c* gene.

## 1. Introduction

Polymerase chain reaction (PCR) based cloning of gene of interest with high GC content is a long recognized problem. PCR is a most sensitive tool and various factors have to be optimized for amplification of gene of interest. Primer is one of the precise control elements in this process. Designing of primers directly influences the result of standardized cloning procedures. High GC content of the gene generates complication during primer designing like mismatch and high annealing temperature, self-dimer formation, and secondary structure. Sometimes, amplification of gene is not routinely achieved by normal PCR techniques. The most prominent problem associated is hairpin loop, which directly interferes during annealing of primers on difficult DNA template that leads to no amplification. Different strategies have been proposed to sort out this problem. Use of DMSO and glycerol was reported to reduce the annealing temperature and denaturation temperature, increase the chances of breakage of secondary structure, and increase the efficiency of amplification [1–5]. The whole genome sequence of* Mycobacterium tuberculosis* was deciphered by Cole et al. [6]. The genes of* M. tuberculosis* are being cloned and expressed in* E. coli* cells in order to identify their possible role in* Mycobacterium* life. The* Mycobacterium* genome has very high GC content (66%) which raised the possibility of hairpin structure in the genomic structure. From genome sequence analysis it was observed that PPE, PE, and PGRS multigene family code for proteins of approximately 110–80 amino acids rich in proline and glutamic acid at N-terminal position. Proline and glutamic acid residues are mainly coded by triplet of GC bases in* Mycobacterium* genome. Most of the genes for membrane proteins of* M. tuberculosis* were rich in GC content at terminal regions. Presence of high GC content increased the annealing temperature beyond the extension temperature (72°C) and also repeated stretches generate the hairpin structure. In such cases, effectiveness and reproducibility of PCR amplification depend on detailed analysis of the possible secondary structures of the oligonucleotide primers as well as formation of self-dimers and cross-dimers with other interrelating oligonucleotides [7]. Though these problems have been considered by several investigators, no systematic details are available to approach this problem.

In an attempt to clone GC rich genes (*Rv0519c* and* Rv0774c* from* M. tuberculosis* and* ML0314c* from* M. leprae*) from* Mycobacterium* sp., we designed primers by using standard method for gene amplification.* Rv0774c* and* Rv0519c* genes demonstrated 100% nucleotide identity in* M. tuberculosis* H37Rv and* M. tuberculosis* H37Ra. Therefore,* M. tuberculosis* H37Ra chromosomal DNA was used as template for amplification of these two genes. We could amplify* Rv0774c* gene, but* Rv0519c* and* ML0314c* genes having high GC content at terminal region were not amplified by standard PCR procedures. Therefore, an attempt has been made in the present investigation to standardize the conditions and ingredients that favor the amplification of GC rich sequences.

## 2. Materials and Methods

### 2.1. Materials

*E. coli* DH5*α* cells and pET-28a were procured from Invitrogen. Taq polymerase and dNTPs were purchased from Fermentas, USA. Restriction enzymes were purchased from New England Biolabs. Kanamycin, Middlebrook media, OADC, and tween 80 were purchased from Hi-Media, India. The strain* Mycobacterium tuberculosis* H37Ra was a kind gift from Director of National Institute of Leprosy and Other Mycobacterial Diseases, Agra, India. Genomic DNA for* Mycobacterium leprosy* was a kind gift from Dr. Mallika Lavania of Stanley Browne Laboratory, the Leprosy Mission, Nandnagri, Shahdara, New Delhi.

### 2.2. *Mycobacterium* Genomic DNA Isolation

*M. tuberculosis* H37Ra strains were routinely cultured for one week on middlebrook, 0.05% Tween 80, enriched with OADC. One week grown* M. tuberculosis *H37Ra cells (1.5 mL) were harvested by centrifugation at 5000 ×g for 20 min. Harvested pellet was resuspended in 400 *μ*L Tris-EDTA buffer (100 mM Tris/10 mM EDTA, pH 8). The cells were lysed by putting the sample in boiling water bath for 5 min followed by cooling on ice for 5 min. Forty microliters of 20 mg/mL lysozyme and 5 *μ*L of proteinase K were added. After 1 h of incubation at 37°C, solution A (56 *μ*L of 10% SDS, 64 *μ*L of CTAB-NaCl) was added to the reaction mixture. After 2 h of incubation at 65°C, proteins were removed by 2-3 times of washing with phenol : chloroform : isoamyl alcohol (25 : 24 : 1). The genomic DNA was precipitated with 0.6 volume of isopropanol at room temperature for 1 h. The precipitated DNA was dried and dissolved in sterile water and stored at −20°C.

### 2.3. Modification of Primers by Codon Optimization

Degeneracy of codon is normally used to overcome the existing problem including change of base at wobble position specific for coding sequence of* Mycobacterium* genome. Designed forward primer of* Rv0519c* contributes about 64% GC content and stretches of GC led to generation of complicated hairpin structure with high value of free energy change Δ*G*. By carefully examining the hairpin structure, introduction of the small base/pair distorted the whole secondary structure. The incorporation we opted in the primer sequence was as follows: guanine (G) base turned into adenosine (A) at wobble position of third codon CGG and thymine (T) to adenine (A) in codon CGT (Table 1). Similarly in reverse primer of* Rv0519c* primer sequence, the adenosine (A) base was turned into thymine (T) at wobble position of last sixth codon CGA.* Mycobacterium leprae* genome sequence also has high guanine and cytosine stretches. Reverse primer sequence of* ML0314c* of* leprae* gene was also modified. Guanine was turned into cytosine at wobble position of TCG codon. The effect of modification was analysed by IDT oligoanalyzer tools.

### 2.4. Amplification of GC Rich Sequences of* Mycobacterium* sp

The primers were designed based on the genomic sequence deposited in GenBank under accession number NC_000962.3 and sequence of* ML0314c* was retrieved from Leprosy database (http://mycobrowser.epfl.ch/leprosy.html). PCR amplifications were performed with Taq polymerase (Fermentas, USA). PCR was carried out in 25 *μ*L reaction volumes containing 75 ng genomic DNA templates, 2.5 mM of dNTP mix, 4 mM MgSO_4_, 1.0 *μ*M of each primer sets, 1 U/*μ*L Taq polymerase, and 1X Tris Buffer containing KCl (Fermentas, USA) and 5% DMSO (v/v). The sequences of both forward and reverse primers are listed in Table 1. These sets of primers were used to clone* Rv0774c* and* Rv0519c* genes from isolated genomic DNA of* M. tuberculosis *H37Ra (Table 1) and* ML0314c* gene from* M. leprae* genomic DNA as follows: denaturation for 4 min at 94°C, then 30 cycles consisting of 50 s at 94°C, 40 s at 63.3°C, and 2 min at 72°C and then 7 min at 72°C for final extension. 16S rDNA was used as positive control. PCR product was analyzed on 1.5% agarose gel and purified with RBC column DNA extraction kit. Amplification of* Rv0519c* and* ML0314c* was not achieved through normal PCR procedures. The gene sequences of* Rv0519c* and* ML0314c* were analysed and the modified primers were reconstructed for the amplification of gene. We followed the above given PCR procedure to amplify the gene with annealing temperatures of 64.5°C and 62°C. PCR products were analyzed on 1.5% agarose gel and purified. To confirm the sequence of amplified product, the purified PCR products for all three genes were sent for sequencing with specific primers.

## 3. Results and Discussion

### 3.1. Problem with Amplification of* Mycobacterium *Gene

**Figure 1:**
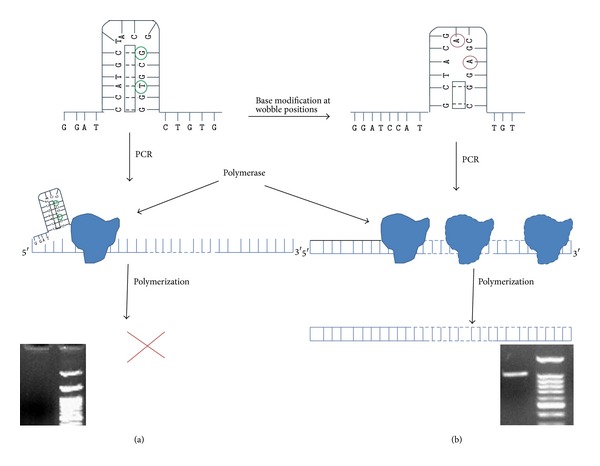
Prediction of secondary structure. (a) Presence of secondary structures in the primers designed by standard primer designing. Secondary structure halts the progression of polymerase. (b) Modification of bases at wobble position distorts secondary structure and allows moving of polymerase. Base modifications are shown in light red circle and the possible hydrogen bond is shown in square box.

**Figure 2:**
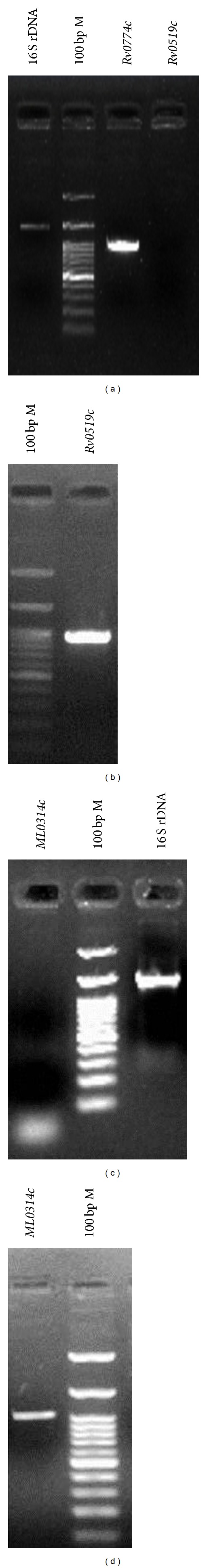
Amplification pattern of 16S rDNA,* Rv0519*, and* Rv0774c* genes from* M. tuberculosis* (Figures 2(a) and 2(b)),* ML0314c* and 16S rDNA from* M. leprae* (Figures 2(c) and 2(d)). (a) Amplification of 16S rDNA,* Rv0519*,* Rv0774c* genes with standard primers, (b) amplification of* Rv0519* with modified primers, (c) amplification of 16S rDNA and* ML0314c* genes with standard primers, and (d) amplification of* ML0314c* with modified primers.

Strategies for the cloning of complicated DNA sequences are of the most significance and it has to be optimised through simple procedures. For PCR based cloning of genes, primer is one of the crucial factors for successful amplification of the genes to be cloned. Length of primer and annealed matches increase the specificity of the reaction but it may not always be an authentic reason to get desired amplicons. With the development of sequencing technology, many tools have been developed to design primers. But the noticeable points with these databases were the variability of primers properties like annealing temperature, prediction of secondary structure, and so forth. Successful amplifications were performed even with primer pairs that were generated through integrative oligoanlyser tools, at annealing temperature close to predicted value (http://eu.idtdna.com/analyzer/applications/oligoanalyzer/). High GC content in the genes of* Mycobacterium species* generate stable secondary structures which often form in the oligos DNA that halt the progression of DNA polymerase during amplification (Figures 1(a) and 1(b)). In the present study, two genes of* M. tuberculosis* were selected and primers were designed. The primer properties were evaluated through different most widely used oligo designing tools such as IDT, Sigma, OC, and manual. Lots of variations were observed in annealing temperature of the same primer sequence. Current study illustrated the need to balance length and melting temperature with respect to GC content of gene at terminal sites, while designing primers for the PCR. It also emphasised the importance of careful investigation of sequences for GC-rich repeats, giving rise to complicated secondary structures, which could reduce the efficiency of amplification [8]. We successfully amplified the* Rv0774c* gene with normal PCR primers and procedure (Figure 2(a), lane 3). On the other hand, no amplification was observed in case of* Rv0519c* gene (GC content 69%) under same reaction conditions (Figure 2(a), lane 4). Attempt had been made to amplify the gene with a long range of annealing temperatures but without success. Application of DMSO and glycerol was also checked with different concentrations (3–10%) with no visible effect on the amplification of the* Rv0519c* gene (data not shown). The16S rDNA was amplified with DNA template (Figure 2(a), lane 1).

On analysis of terminal regions of the* Rv0774c* and* Rv0519c* genes, we observed very high GC content (Table 1). The presence of stable hairpin structures was not observed in case of* Rv0774c*, despite the high GC content while the high GC content at terminal region of* Rv0519c* gene led to formation of strong hairpin loop structure formation during normal primer designing procedure (Table 1). In normal forward primer the value of Δ*G* is more towards negative side (Δ*G* = −3.67 Kcal/mol), and a complicated hairpin structure formation was observed in case of standard reverse primer used for amplification of* Rv0519c* gene (Table 1). Such high −Δ*G* value and complicated hairpin structure might create problem during amplification procedures. Thus, it was essential to decrease the GC content and change of corresponding nucleotides of oligos according to the wobble hypothesis approach. Therefore, we designed the primers by using the degeneracy of codon and checked the hairpin loop formation by integrative DNA-oligoanalyser. All the introduced modifications at wobble position were analysed on IDT tools for their effectiveness to distort hairpin. Out of five changes, one by one and together, we observed that single/double point changes were able to distort the whole secondary structure (data not shown). We introduced small change at nucleotide level, starting with codon CGG to CGA and CGT to CGA during designing of forward primer sequence while single nucleotide change CGT to CGA in case of reverse primer distorted the possible hairpin structure. Introduction of small changes at nucleotide level during primer designing reduced the chances of hairpin loop formation (Δ*G* = −0.31 Kcal/mol), resulting in sharp amplification of* Rv0519c* gene through normal PCR procedures (Figure 2(c), lane 2). Similar problem was observed in amplification of* ML0314c* gene (GC content, 60%) from* M. leprae *while 16S rDNA was successfully amplified from* M. leprae* genome (Figure 2(b), lane 3). Primers designed for* ML0314c* gene from normal procedures demonstrated mismatch annealing temperature as well as strong hairpin loop structure (Table 1). Similar approach was used to modify the forward and reverse primers for* ML0314c* gene. This problem was also tackled successfully by changing nucleotide as well as addition of nucleotide at terminal position, upstream to the restriction sites to sort out the annealing mismatch between the primers. Δ*G* for reverse primer was changed from −1.45 to −1.19 Kcal/mol. The single nucleotide modification at wobble position TCG to TCC distorted the whole hairpin structure. The strategy was successfully confirmed by amplification of* ML0314c* gene (Figure 2(d), lane 1) from* M. leprae* genome. Sequencing of PCR products confirmed the amplification of specific genes.

**Figure 3:**
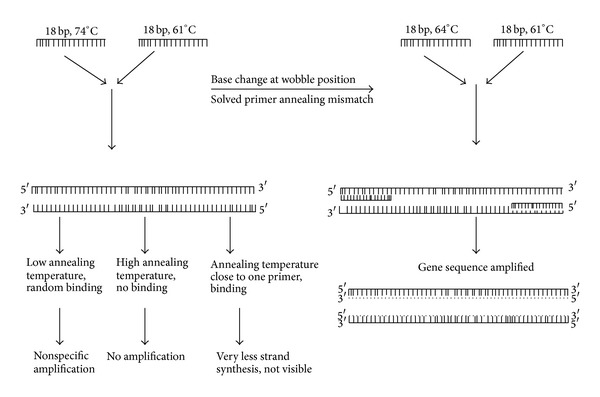
Schematic presentation of the annealing primer mismatch and use of wobble hypothesis approach to sort out this problem.

A major benefit of this protocol was to resolve the problem of Tm mismatch as well as existence of secondary structure in the primer pairs of high GC rich sequences. By using this approach we could adjust the Tm mismatch without increasing the length of primers which may be responsible for dimerization. The high Tm of primers, which become a setback in carrying out PCR, was easily solved by this method (Figure 3). This strategy might work successfully for amplification of PPE, PE, and PGRS protein of* M. tuberculosis* containing large number of proline and aspartate residues that are mostly coded by repeats or stretch of G or C, which increased the chance of hairpin loop formation and annealing mismatch during standard PCR primer designing.

## 4. Conclusion

By using the wobble hypothesis approach in primer designing, we were able to tackle the ongoing amplification problem associated with complicated gene of* Mycobacterium* with high GC content. In this approach a single modification at base level distorted the secondary structure and resolved the primer Tm mismatch. The resulting gene sequence is different slightly from the canonical sequence of the reference genome without modification in the amino acid sequence of the protein.

## Figures and Tables

**Table 1:** Comparison of standard (normal) and modified primer sequences and its secondary structure for amplification of GC rich sequences.

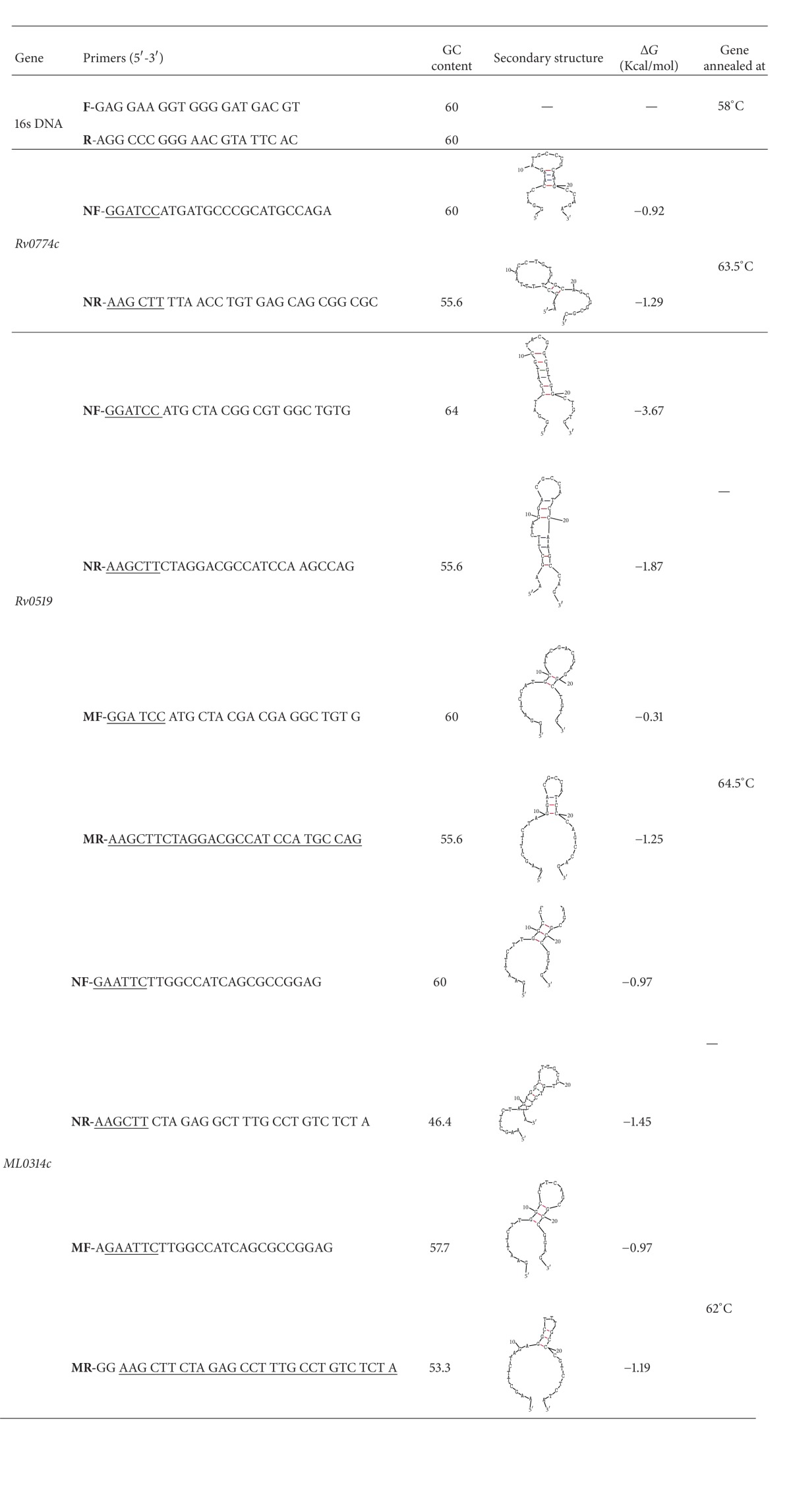

NF: normal forward; NR: normal reverse; MF: modified forward; MR: modified reverse.

Data evaluation by oligo-analyzer tools on IDT: integrative DNA technology.
